# Effects of Exercise Training on Heart Rate Variability in Healthy Adults: A Systematic Review and Meta-analysis of Randomized Controlled Trials

**DOI:** 10.7759/cureus.62465

**Published:** 2024-06-16

**Authors:** Youssra Amekran, Abdelkader Jalil El hangouche

**Affiliations:** 1 Department of Physiology, Faculty of Medicine and Pharmacy, Abdelmalek Essaadi University, Tangier, MAR

**Keywords:** meta-analysis, healthy adults, exercise, heart rate variability, autonomic nervous system

## Abstract

This meta-analysis was conducted to investigate the effects of exercise training on heart rate variability (HRV) parameters associated with the autonomic nervous system (ANS) activity. Randomized controlled trials (RCTs) involving healthy adults (aged ≥ 18 years) were included. We searched PubMed, Scopus, Web of Science, and EBSCO databases to identify relevant studies. A random-effects meta-analysis was performed using the standardized mean difference (SMD) and 95% confidence interval (CI). Sixteen RCTs with a total of 623 participants were selected for the final analysis. The analysis showed that exercise training improved the standard deviation of normal-to-normal intervals (SDNN) (SMD: 0.58 (0.16, 1.00); p = 0.007), the root mean square of successive differences in heart period series (RMSSD) (SMD: 0.84 (0.36, 1.31); p = 0.0005), and the absolute power of high-frequency band (HF) (SMD: 0.89 (0.27, 1.51); p = 0.005) parameters compared to the control group. Analysis of the moderator variables showed that the effect of exercise on HRV indices may be influenced by sex, age, and type of exercise used, specifically in HF band, absolute power of low-frequency band (LF), and LF/HF ratio parameters. Despite the limited number of existing RCTs related to the subject, the results suggest that exercise training enhances HRV parameters associated with vagal-related activity (RMSSD and HF) and both sympathetic and parasympathetic activities (SDNN). This study overcomes the lack of meta-analyses on the effects of exercise training on autonomic modulation among healthy adults and may bridge the gap in understanding the potential physiological underpinnings of the acknowledged positive health benefits of exercise.

## Introduction and background

The autonomic nervous system (ANS) is the part of the peripheral nervous system that regulates involuntary functions, including heart rate, blood pressure, respiration, and digestion. It consists of two primary branches, the sympathetic and parasympathetic nervous systems (SNS and PNS) [1]. Given the complexity of the ANS, various tests have been developed to assess the function of ANS branches in diverse research and clinical settings, including the analysis of heart rate variability (HRV) [2]. Heart rate is the number of heartbeats per minute, and HRV is the variation in time between RR-intervals (RR-intervals, the time elapsed between two consecutive R waves of the electrocardiogram (ECG) QRS complex) [3]. HRV is an important physiological marker that reflects vagal and sympathetic nerve activity [4-6]. It can be assessed using a range of analytical methods, although the most commonly used are time-domain and frequency-domain (power spectral density) analyses [7]. Standards for HRV measurement, interpretation, and clinical application of these methods were first established by the European Society of Cardiology (ESC) and the North American Society of Pacing and Electrophysiology (NASPE) [8].

Time-domain analysis of HRV quantifies variability in the interbit interval. The most common time-domain parameters used are the standard deviation of normal to normal (NN) intervals (SDNN), root mean square of successive differences in the heart period series (RMSSD), and percentage of adjacent NN intervals varying by more than 50 milliseconds (pNN50). The SDNN and RMSSD indices are measured in milliseconds, and pNN50 is a percentage (%) [8]. Frequency-domain HRV, also known as power spectral density analysis, describes the distribution of heart rate oscillations in four frequency bands: ultra-low-frequency (ULF: ≤0.003 Hz), very-low-frequency (VLF: 0.0033-0.04 Hz), low-frequency (LF: 0.04-0.15 Hz), and high-frequency (HF: 0.15-0.4 Hz). The sum of these four spectral bands is known as the total power of inter-beat variability [8,9]. Frequency-domain HRV indices can be presented as absolute values (millisecond squared; ms²) or normalized unit values (nu) [9]. Henceforth in this paper, absolute values are noted LF and HF, and normalized values LFnu and HFnu. HRV measurements can be obtained through long-term (24h), short-term (ST, ~5 min), and ultrashort-term (UST, < 5 min) recordings [10]. In time-domain analysis, the SDNN provides an estimate of both sympathetic and parasympathetic activities and shows a high correlation with the ULF, VLF, and LF bands. RMSSD reflects vagal tone activity and is closely associated with HF. Finally, the pNN50 index is correlated with RMSSD and HF and is thought to reflect parasympathetic activity [10-12]. In frequency domain analysis, while LF has traditionally been thought to reflect cardiac sympathetic outflow, more recent studies have cast doubt on this hypothesis and have instead suggested that both sympathetic and parasympathetic outflows contribute to LF power [7,12]. Regarding the HF parameter, there is more consensus between studies regarding its origin than the LF band, as these studies consistently support the influence of PNS activity on HF [10]. HF is commonly referred to as the respiratory band because it is aligned with the heart rate variations related to the respiratory cycle [10]. Finally, HRV studies consider the ratio between the LF and HF bands (LF/HF) as an index reflecting the balance between sympathetic and parasympathetic activity [12]. In recent decades, HRV has been used to recognize both healthy and diseased states. ANS imbalances, indicated by increased SNS and decreased PNS activity, are linked to the pathogenesis and development of various diseases, including hypertension and cardiovascular diseases, as well as an increased risk of sudden death [13-16]. Furthermore, enhanced HRV indicates the cardiac system’s ability to adapt to intrinsic and extrinsic variations such as stress and physical exercise [17]. Mounting guidelines recommend adopting lifestyle improvements, including physical activity and exercise, for the prevention and treatment of many cardiovascular and other chronic diseases [18,19]. Several studies have reported that exercise training triggers neuromodulation and can significantly enhance the sympathovagal balance of the ANS in sedentary individuals, athletes, and diseased populations [20-22].

Aerobic exercise training, defined as any activity involving large muscle groups and relying on aerobic metabolism that uses oxygen to extract energy, has been reported to be effective in modulating autonomic nerve activity and improving imbalances in hypersympathetic or decreased parasympathetic activity [23,24]. Long-term aerobic training has been associated with enhanced resting vagal-related HRV indices in adults [25-28]. In contrast, short-term moderate-intensity aerobic training and high-intensity interval training programs failed to significantly affect HRV parameters in physically inactive adults [29]. The effects of anaerobic exercise, defined as intense and short-duration physical activity fueled by energy sources within the contracting muscles independently of the use of inhaled oxygen, on the modulation of the ANS have not yet been extensively investigated. However, it has been suggested that anaerobic training appears to have no effect on HRV in healthy young adults, while it helps improve parasympathetic modulation in middle-aged adults with autonomic dysfunction [28,30].

The literature on exercise training-induced effects on autonomic modulation reports controversial results, necessitating a comprehensive and critical evaluation of these findings. Discrepancies between studies on exercise and HRV may be attributed to the use of different exercise modalities and variations in HRV measurement methodologies, which complicates the understanding of the effect of exercise on HRV and decision-making, relying only on individual study outcomes and expert opinions. While the meta-analysis approach is regarded as providing high-level evidence, there is a limited number of meta-analyses available on the topic, especially among healthy populations. Hence, the present systematic review and meta-analysis, performed on data gathered from randomized controlled trials (RCTs), aimed to assess the effect of exercise training on time- and frequency-domain HRV in healthy adults. Moreover, the analysis will consider participants and intervention characteristics to determine potential influencing factors.

## Review

Methods

This systematic review and meta-analysis followed the Preferred Reporting Items for Systematic Reviews and Meta-analyses (PRISMA) [31] and Cochrane Handbook for Systematic Review interventions’ guidelines [32]. The protocol is registered in the International Prospective Register of Systematic Reviews (PROSPERO) with identification number CRD42023459682.

Search strategy

We systematically searched PubMed, Scopus, Web of Science, and EBSCO databases using a strategy combining terms relating to exercise, ANS activity, and HRV: exercise, “exercise training,” training, “physical activity,” “aerobic exercise,” “aerobic training,” “endurance exercise,” “endurance training,” “anaerobic exercise,” “anaerobic training,” “resistance exercise,” “resistance training,” “high-intensity interval training,” “heart rate variability,” HRV, “autonomic nervous system,” “autonomic function,” “autonomic activity,” “autonomic modulation,” “autonomic regulation,” “cardiac autonomic modulation,” “cardiac autonomic control,” “cardiac autonomic regulation,” “sympathetic nervous system,” “sympathetic function,” “sympathetic activity,” “parasympathetic nervous system,” “parasympathetic function,” “parasympathetic activity,” “vagal function,” “vagal activity,” and “vagal tone.” Filters limiting research to RCTs and English, French, and Spanish publications were used when applicable. No date limitation was used. The reference lists of the previous articles were manually searched to ensure the inclusion of potentially relevant studies. The search was performed on October 25, 2023.

Eligibility criteria

Eligibility criteria were established according to the PICOS (participants, intervention, comparison, outcomes, and study design) principle.

Participants

Healthy adults aged 18 years and above with no restrictions based on sex.

Intervention

Aerobic or anaerobic exercise training programs lasting 4 or more weeks.

Comparison

Comparison between the experimental group (received exercise training intervention) and control group ( no intervention).

Outcomes (O)

Studies should report measurement of ANS activity using time-domain HRV indices (SDNN, RMSSD, and pNN50) and frequency-domain indices (LF, LFnu, HF, HFnu, and LF/HF ratio), in addition to changes in HRV parameters (pre- and post-intervention values).

Study Design (S): RCTs

Studies not evaluating the effects of exercise training intervention on the ANS activity using HRV, involving participants under the age of 18 years or with diseases or medical conditions, examining exercise training programs lasting less than 4 weeks, and studies lacking standardization or a control group in their design were excluded.

Data extraction

The authors conducted the literature search, and they independently removed duplicates and reviewed titles, abstracts, and full-text to assess the suitability of articles based on the selection criteria, as recommended by the Cochrane Handbook guidelines. When necessary, disagreements regarding the eligibility of some of the identified trials were discussed and resolved by consensus. From the publications identified for inclusion, the following study data were extracted: study characteristics (first author, date of publication, country, and sample size), participant characteristics (age and sex), intervention characteristics (type of intervention (aerobic or anaerobic exercise or combined), duration of intervention, exercise frequency, number of exercise sessions, duration of each session, and intensity), methodological aspects of the outcome measurement (recording device, recording position, if measurement is carried out in several positions, results from the supine position was preferred and extracted), assessment length, and respiration (spontaneous or controlled), the outcome being assessed (mean and standard deviation (SD) or standard error (SE) of time- and frequency-domain HRV parameters), and their reported changes from baseline to the end of the intervention.

The extracted data on study characteristics and outcomes were transferred into the Review Manager software (RevMan 5.4; Cochrane Collaboration, Oxford, UK).

Risk of bias assessment

Methodological quality was independently assessed by the authors using the revised version of the Cochrane risk of bias tool (Rob2) [33] and the tool for the assessment of study quality and reporting in exercise (TESTEX) [34]. Rob2 is a domain-based critical assessment of the following core risk of bias domains: randomization process, deviations from intended interventions, missing outcome data, outcome measurement, and selection of reported results. For each study, the risk of bias in each domain was described as low, with some concerns, or high risk, and the overall risk of bias in the study was obtained through the tool’s algorithm. The TESTEX scale consists of 12 items with one point scored if the respective criterion was met. The maximum score is 15 (Items 6 and 8 have three and two associated questions, respectively). Methodological quality was assessed based on the total score, and judged as excellent (12-15), good (9-11), fair (6-8), or poor (<6). Publication bias was assessed using funnel plot asymmetry.

Statistical analysis

Statistical analyses were conducted to determine the effect of exercise training on HRV using the Review Manager software (RevMan 5.4; Cochrane Collaboration, Oxford, UK). Owing to the differences between the included studies, a random-effects model was used. Given that the outcome variables were continuous, mean and SD were extracted for each included study. If SEs were provided as dispersion measures, they were converted into SDs ($SD = SE \times \sqrt{n}$) prior to analysis. The mean change ($\text{Post} - \text{Pre-intervention}$) in the outcomes in each of the intervention and control groups with SD was determined. When SD is not available in the eligible studies, it is obtained using the formula \begin{equation*} SD = \sqrt{SD_{\text{baseline}}^2 + SD_{\text{final}}^2 - (2 \times \text{Corr} \times SD_{\text{baseline}} \times SD_{\text{final}})} \end{equation*} (with Corr: correlation coefficient), according to the Cochrane Handbook recommendations [32]. The following correlation coefficients were obtained for SDNN, RMSSD, HF, HFnu, and LFnu: 0.59, 0.87, 0.79, 0.61, and 0.5, respectively. As the correlation coefficients calculated to derive SDs for changes from baseline in the pNN50, LFnu, and LF/HF indices were less than 0.5, final values (post-intervention) were compared between the intervention and control groups. This is in accordance with the Cochrane Handbook guidelines stating that there is no value in using changes from baseline when the imputed coefficient is less than 0.5; therefore, analyzing the final values is deemed more precise [32]. SDNN and RMSSD values expressed in ms, and LF and HF in absolute and normalized units, separately, (LF, HF, LFnu, HFnu, and HFnu), were considered in the pooled analyses.

To avoid the unit-of-analysis error for trials with multiple intervention arms and a common single control group, the sample size for the control group was weighted by the number of groups and participants treated, and the means and standard deviations remained unchanged.

The overall effect of exercise training versus no intervention was assessed using the standardized mean difference (SMD) with a 95% confidence interval (CI) as the effect size (ES) index. Subgroup analyses were performed to identify potential effect modifiers related to the population and intervention characteristics. The following characteristics were included in the analyses: age (< 40 and ≥ 40 years), sex (male, female, and both sexes), type of intervention (aerobic, anaerobic, and combined exercise training), and intervention duration (< 12 and ≥ 12 weeks).

We explored the heterogeneity between the included studies qualitatively through visual inspection of forest plots and quantitatively using the Chi² test of heterogeneity in conjunction with the I² statistic. A statistically significant result is indicative of substantial heterogeneity. I² values indicate no heterogeneity (0-25%), moderate heterogeneity (30-60%), substantial heterogeneity (50-90%), and considerable heterogeneity (>75%) [32]. Potential sources of heterogeneity were explored using subgroup analyses and sensitivity analysis. All statistical analyses were two-tailed and the level of significance was set at p ≤ 0.05.

Results

**Figure 1:**
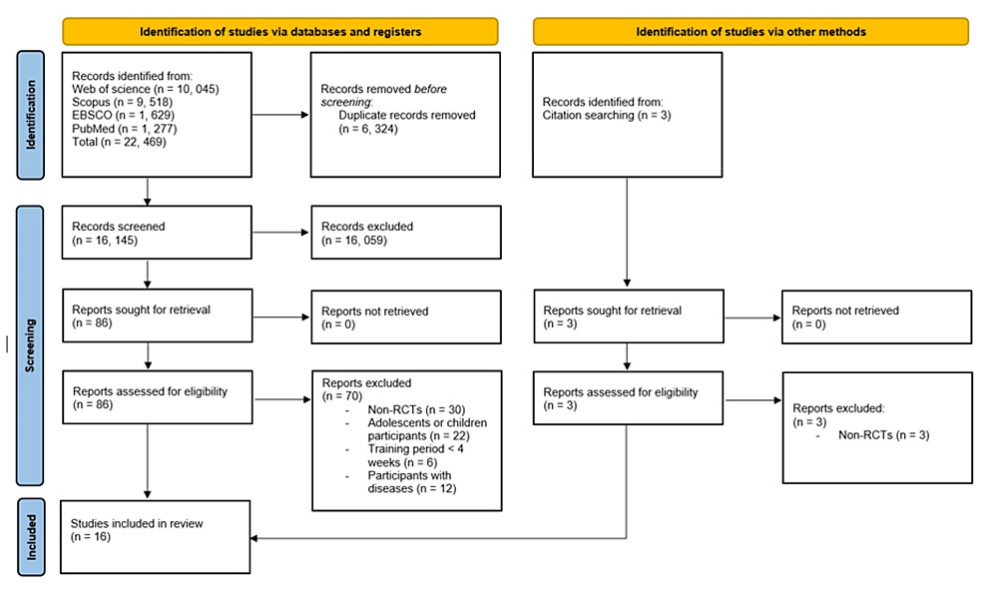
PRISMA flow diagram summarizing the study selection process. The figure was drawn by the authors of this article.

Our research strategy using electronic databases retrieved 16,145 studies after removing duplicates. After screening titles and abstracts, 86 trials were selected for full-text review, of which 70 were excluded due to irrelevant methodological, population, or intervention characteristics that did not meet our eligibility criteria. Finally, 16 RCTs were included in the analysis. A description of the study selection process is presented in Figure 1.

Table 1 presents an overview of the included studies, participants, intervention, and outcome characteristics. The studies were conducted in different geographic locations (6 in Brazil, 2 in Australia, 2 in Taiwan, 1 in each of Canada, Iran, Japan, New Zealand, Thailand, and the United Kingdom). Out of the 16 studies, seven recruited male participants [15,35-40], four recruited female participants [41-44], and the remaining five studies enrolled participants of both genders [45-49]. A total of 623 participants were included in this systematic review and meta-analysis (346 in the training group and 277 in the no-training control group). The sample size of the individual studies ranged from 20 to 59. Five studies included more than one intervention group [15,36,39,40,49]; however, because comparisons were independent, the unit-of-analysis error was overcome [32]. The mean age of participants ranged from 19.2 ± 0.8 to 68 ± 5.5 years. No differences were reported between the intervention and control groups in any of the included studies (except for Rezende Barbosa et al. [44], where the intervention group and control group participants were significantly different in terms of age).

The training program interventions included aerobic [15,35,36,39-41,43,49], anaerobic [38,39,42,45,48,49], and combined training sessions [37,44]. The mean intervention length was 10.4 weeks ranging from 4 weeks [45] to 8 months [49]. In the majority of studies, participants trained three times weekly [15,35,37,39-41,45-49], in four studies, they carried out two sessions per week [38,42-44] and in one study, participants trained 6 days per week [36]. Regarding the intensity of exercises used, it ranged, in the studies that reported this information, between 40% and 100% of heart rate reserve (HRR), heart rate peak (HR peak), maximum oxygen consumption (VO2 max), or of repetition maximum (1RM).

HRV was measured using an ECG [15,38,39,41-43,45-47], and Polar monitors [35-37,40,44,48,49]. The recording lasted 5 min [15,39,41,45,49], 10 min [46], 20 min [35,40,42,43,48], 30 min [37,44], and 24h [36,38,47]. While six studies did not report recording body position [35,36,38,39,41,47], the remaining 10 studies [15,37,40,42-45,47,48,49] performed the measurements in the supine position. If a study carried out the measurement in different positions, the results from the supine position were analyzed. Participants breathed spontaneously during HRV assessment in eight studies [15,35,36,38,40,44,46,49], and controlled breathing was used in only two studies [39,48], and the remaining six studies did not disclose this information [37,41-43,45,47]. The time-domain HRV parameters reported by the included studies were SDNN [35,36,39-42,44,48,49], RMSDD [15,35,37,39-42,44,48], and pNN50 [37,39,41,42,46]. For the frequency-domain HRV, we considered LF and HF parameters presented in absolute values [35,37,40,42,44,45,47-49], normalized units values [15,35,36,38,41-45,48], and LF/HF ratio [15,36,38,40-45,47,48].

**Table 1:** Descriptive characteristics of the studies included in the meta-analysis. A-HITG: high-intensity training with active recovery group; ATG: aerobic training group; CG: control group; CHG: control high resting cardiac vagal modulation group; CLG: control low resting cardiac vagal modulation group; ECG: electrocardiogram; HF: power of the high-frequency band; HFnu: normalized unit of power of the high-frequency band; HIIE, high-intensity intermittent exercise; HR: heart rate; HRmax: Maximum heart rate; HRR: heart rate reserve; HRV: heart rate variability; HTG: high-volume training group; HVMITG: high-volume moderate-intensity training group; LF: power of the low-frequency band; LFnu: normalized unit of power of the low-frequency band; LVHITG: low-volume high-intensity training group; MICTG: moderate-intensity continuous training group; ms: milliseconds; MTG: moderate volume training group; nu: normalized unit; P-HITG: high-intensity training with passive recovery group; pNN50: percentage of adjacent NN intervals varying by more than 50 milliseconds; RM: repetition maximum; RMSSD: root mean square of successive RR-intervals differences; RTG: resistance training group; SD: standard deviation; SDNN: standard deviation of NN intervals; SG: sham group; TG: training group; THG: training high resting cardiac vagal modulation group; TLG: training low resting cardiac vagal modulation group; VO_2_max: maximum oxygen consumption ↔ indicates no change; ↑ indicates increase, ↓ indicates decrease; *significant changes (p-value ≤ 0.05) for post- baseline comparison; + p-value for post-baseline comparison not reported.

Author, year, and country	Sample size	Groups, mean age	Exercise training (Exercise type, length, total number of sessions, frequency, intensity, and duration of training program, when available)	Outcome (HRV) measurement (Recording device, duration, position, and respiration, when available)	HRV changes from baseline		
						Cavina et al. 2021, Brazil [35]	Total: 54, mean age ± SD: 26.58 ± 4.4 years, female Gender (%): 0	TG (n = 28), 27.11 ± 3.78	Pilates training, 60 min, 36 sessions, 3 × per week, Duration: 12 weeks	Polar V800 (Polar Electro Oy), 20-min, spontaneous breathing	↑SDNN*		
↑RMSSD										
↑LF										
↑LFnu*										
↑HF										
↓HFnu*										
CG (n = 26), 26.00 ± 4.63	↓SDNN									
	↓RMSSD									
	↓LF									
	↓HF									
	↓LFnu									
	↓HFnu									
Cheema et al. 2013, Australia [46]	Total: 37, 38 ± 12, female Gender (%): 81.08	TG (n = 18), 37 ± 12	Yoga program, Yoga program, 50 min, 3 × per week, Duration: 10 weeks	ECG, 10-minute period, supine position, spontaneous breathing	↓pNN50 +		
		TG (n = 19), 39 ± 13			↑pNN50 +		
						Chu et al. 2015, Taiwan [43]	Total: 52, 26.21 ± 5.72, female Gender (%): 100	TG (n = 26), 27.58 ± 6.12	Yoga program, 60-minute, 2 × per week, Duration: 8 weeks	ECG, 20-min period, supine position	↑LFnu +		
↓HFnu +										
↑LF/HF +										
CG (n = 26), 24.85 ± 5.17	↑LFnu									
	↓HFnu									
	↑LF/HF									
Collins et al. 2023, New Zealand [39]	Total: 59, female Gender (%): 0	MICTG (n = 15), 51.1 ± 5.7	Moderate and high interval train, moderate and high interval training (cycle ergometer), 3 × per week, MICTG: 50 – 60 min of continuous cycling, 3 × per week, 60–70% of HRmax, HITGs: 30-s work intervals interspaced with 2.5 min of active or passive recovery, 85% HRmax. Duration: 12 weeks in (cycle ergometer), 3 × per week	ECG, 5-min period, controlled breathing: 12 breaths/min	↑SDNN*		
					↑RMSSD		
					↓pNN50		
		P-HITG (n = 15), 47.3 ± 5.1			↑SDNN*		
					↑RMSSD		
					↑pNN50		
		A-HITG (n = 15), 49.1 ± 5.3			↑SDNN		
					↑RMSSD		
					↑pNN50		
		CG (n = 14), 51.2 ± 7			↑SDNN		
					↓RMSSD		
					↓pNN50		
Decaux et al. 2022, United Kingdom [45]	Total: 30, 30.2 ± 8.4, female Gender (%): 50	TG (n = 10), 31.4 ± 6	Isometric exercise training, 4 × 2-min bouts separated by 2-min rest intervals, 3 × per week, 95% peak HR, duration: 4 weeks	ECG, 5-min, supine position	↓LF		
					↓LFnu*		
					↑HF		
					↑HFnu*		
					↓LF/HF		
		CG (n = 10), 28.3 ± 5.6			↑LF		
					↑LFnu		
					↓HF		
					↓HFnu		
					↑LF/HF		
		SG (n = 10), 29.4 ± 7.8			↑LF		
					↑LFnu		
					↑HF		
					↓HFnu		
					↑LF/HF		
Duarte et al. 2015, Brazil [15]	Total: 40, 19.2 ± 0.8, female Gender (%): 0	TLG (n = 11), 19.2 ± 1.0	Aerobic training, 40 min, 3 × per week, 75%–85% HRR, duration: 12 weeks	ECG, 5-min, supine position, spontaneous breathing	↑RMSSD*		
					↓LFnu		
					↑HFnu*		
					↓LF/HF		
		THG (n = 11), 19.5 ± 1.2			↑RMSSD		
					↑LFnu		
					↓HFnu		
					↑LF/HF		
		CLG (n = 9), 19.1 ± 0.3			↑RMSSD		
					↔LFnu		
					↓HFnu		
					↑LF/HF		
		CHG (n = 9), 18.9 ± 0.6			↓RMSSD		
					↑LFnu		
					↓HFnu		
					↑LF/HF		
Gambassi et al., 2016, Brazil [42]	Total: 26, 65 ± 3, female Gender (%): 100	TG (n = 13), 35± 10	Dynamic resistance training, 2 × per week, duration: 12 weeks	ECG, 20-min, supine position	↑SDNN*		
					↑RMSSD*		
					↑PNN50*		
					↑LF		
					↓LFnu*		
					↑HF*		
					↑HFnu*		
					↓LF/HF*		
		CG (n = 13), 35± 10			↓SDNN		
					↑RMSSD		
					↑PNN50		
					↑LF		
					↓LFnu		
					↑HF		
					↑HFnu		
					↑LF/HF		
Heydari et al., 2013, Australia [37]	Total: 30, 24.9 ± 4.3, female Gender (%): 0	TG (n = 15)	Supervised HIIE training (cycle ergometer), 5-min warm-up + 20 min of 8-s sprint + 12-s recovery + 5-min cool-down, 3 × per week, 80–90 % of HR max, duration: 12 weeks	Polar RS800CX (Polar Electro Oy), 30-min	↑RMSSD +		
					↑PNN50 +		
					↑LF +		
					↑HF +		
		CG (n = 15)			↓RMSSD +		
					↓PNN50 +		
					↑LF +		
					↓HF +		
Kanegusuku et al., 2015, Brazil [38]	Total: 25, female Gender (%): 0	TG (n = 12), 64 ± 4	High-intensity progressive resistance training, 7 exercises, 2–4 sets, 2 × per week, 10–4 RM, duration: 4 months	ECG, 24-h, spontaneous breathing	↑LFnu +		
					↓HFnu +		
					↑LF/HF +		
		CG (n = 13), 63 ± 4			↑LFnu +		
					↓HFnu +		
					↓LF/HF +		
Rezende Barbosa et al., 2016, Brazil [44]	Total: 29, female gender (%): 100	TG (n = 13), 23 ± 2.51	Functional training, 2 × per week, 30%-100% of the 1RM, duration: 12 weeks	Polar S810i (Polar Electro Oy), 30-min, supine position, spontaneous breathing	↑SDNN +		
					↑RMSSD +		
					↑LF +		
					↑LFnu +		
					↑HF +		
					↓HFnu +		
					↓LF/HF +		
		TG (n = 16), 20.56 ± 1.03			↑SDNN +		
					↑RMSSD +		
					↓LF		
					↑LFnu+		
					↑HF		
					↓HFnu +		
					↓LF/HF +		
Shen and Wen, 2013, Taiwan [41]	Total: 44, 58.48 ± 0.53, female gender (%): 100	TG (n = 22), 57.86 ± 0.64	Supervised group-based step aerobic exercise, 90 min, 3 × per week, 75–85 % HRR. Duration: 10 weeks	ECG, 5-min	↓RMSSD		
					↓SDNN*		
					↓ pNN50*		
					↓LFnu*		
					↑HFnu*		
					↓LF/HF*		
		CG (n = 22), 59.10 ± 0.83			↑RMSSD		
					↓SDNN		
					↑ pNN50		
					↓LFnu		
					↑HFnu		
					↓LF/HF		
Shiotani et al., 2009, Japan [47]	Total: 35, 22±2, female gender (%): 62.9	TG (n = 16), 26.00 ± 4.63	Aerobic exercise (ergometer cycling), 35 min, 3 × per week, 60%. Duration: 8 weeks	Holter recorder, 24-h	↑HF		
					↓LF/HF*		
		CG (n = 19), 26.00 ± 4.63			↑HF		
					↓LF/HF		
Soltani et al., 2021, Iran [40]	Total: 45, 42 ± 5.7, female gender (%): 0	HVMITG (n = 15), 42.5 ± 6.2	High- and low-volume moderate-intensity training, HVMITG: 45–60 min running, 3 × per week, 50–60% of VO2max, LVHITG: 25–40 min running, 3 × per week, 70–85% of VO_2_max. Duration: 12 weeks	Polar V800 (Polar Electro Oy), 20-min period, supine position, spontaneous breathing	↑SDNN*		
					↑RMSSD*		
					↓LF*		
					↑HF*		
					↓LF/HF*		
		LVHITG (n = 15), 42.2 ± 5			↑SDNN*		
					↑RMSSD*		
					↓LF*		
					↑HF*		
					↓LF/HF*		
		CG (n = 15), 41.5 ± 5.6			↓SDNN		
					↑RMSSD		
					↑LF		
					↓HF		
					↑LF/HF		
Songsorn et al., 2022, Thailand [48]	Total: 21, female gender (%): 54.5	TG (n = 10), 22 ± 0.8	Whole-body HIIT, (burpees, mountain climbers, jumping jacks, and squats), 10 min, 3 × per week, at a maximal effort. Duration: 6 weeks	Polar V800 (Polar Electro Oy), 20-min period, supine position, controlled breathing at 12 breaths/min	↑SDNN*		
					↑RMSSD*		
					↑LF		
					↓LFnu		
					↑HF		
					↑HFnu		
					↓LF/HF		
		CG (n = 11), 21.7 ± 0.8			↓SDNN		
					↑RMSSD		
					↓LF		
					↓LFnu		
					↑HF		
					↑HFnu		
					↓LF/HF		
Tulppo et al., 2003, Canada [36]	Total: 46, female gender (%): 0	MTG (n = 19), 35± 10	Aerobic training (walking and jogging), 30-min (for moderate volume training group) and 60-min (for high-volume training group), 6 × per week, 70–80% of Hrmax. Duration: 8 weeks	Polar (Polar Electro Oy), 24-h, spontaneous breathing	↑SDNN*		
					↓LFnu*		
					↑HFnu*		
					↓LF/HF*		
		HTG (n = 16), 35± 10			↑SDNN*		
					↓LFnu*		
					↑HFnu*		
					↓LF/HF*		
		CG (n = 11), 36± 11			↑SDNN		
					↓LFnu		
					↑HFnu		
					↓LF/HF		
Wanderley et al., 2013, Brazil [49]	Total: 50, 68 ± 5.5, female gender (%): 78	ATG (n = 20), 69.9 ± 5.7	Aerobic and resistance training: Aerobic training group: 50 min (10-min warm-up, 30 min walking aerobic exercise, stepping and dancing, and 10-min cool-down), 3 × per week, 50–80% of HR reserve. Resistance training group: 50 min (10-min warm-up), and low-intensity exercises (walking, biking), 30-min resistance exercises (leg press, chest press, leg extension, seated row, seated leg curl, abdominal flexion, biceps curl, low-back extension, and triceps extension), and 10-min cool-down), 3 × per week, 50–80% of 1RM, duration: 8 months	Polar (Polar NV vantage), 5-min period, supine position, spontaneous breathing	↓SDNN		
					↑HF		
		RTG (n = 11), 67.3 ± 4.9			↑SDNN		
					↑HF		
		CG (n = 19), 67.8 ± 5.5			↑SDNN		
					↑HF		

**Figure 2:**
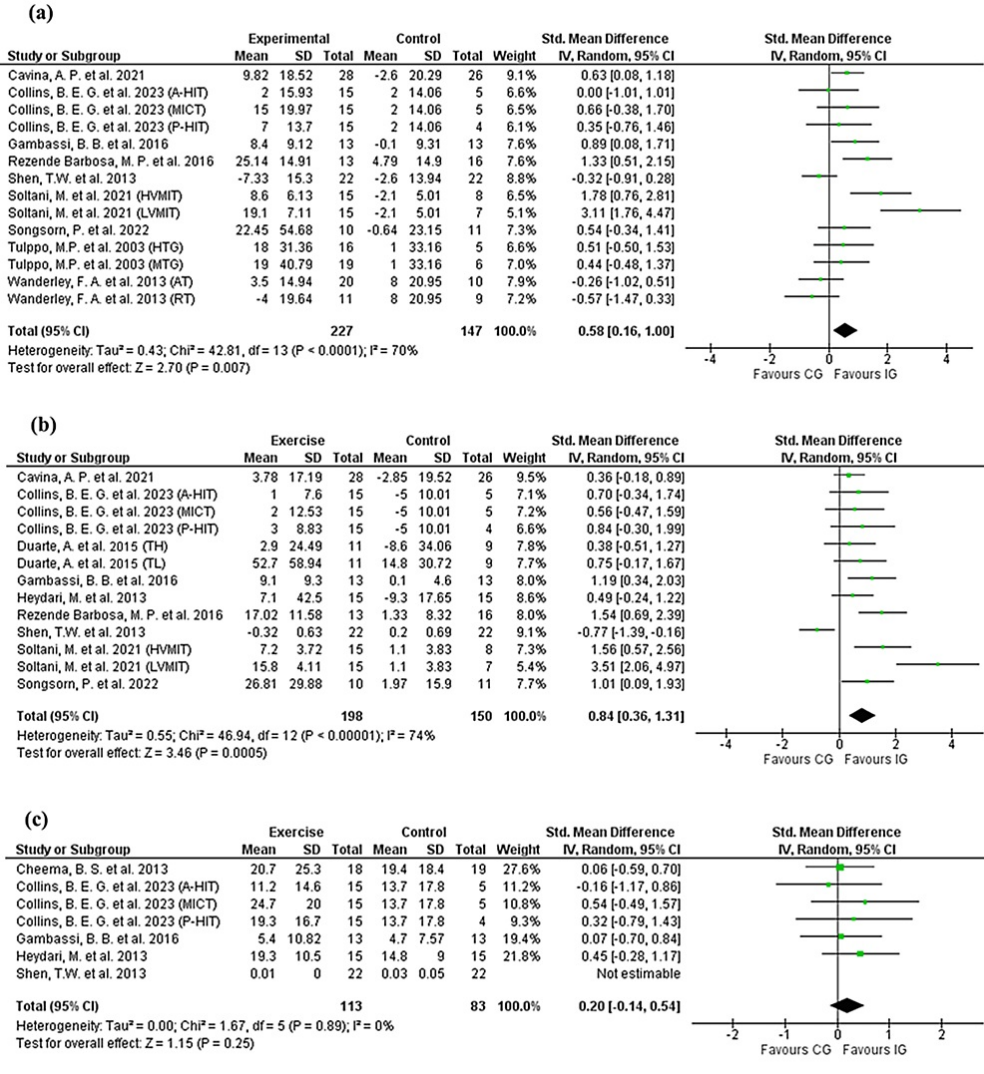
Forest plots of the pooled standardized mean difference (SMD) between exercise and control groups for time-domain HRV indices. a) SDNN (ms) [35,36,39-42,44,48,49], (b) RMSSD (ms) [15,35,37,39-42,44,48], (c) pNN50 [37,39,41,42,46] A-HIT: high-intensity training with active recovery; AT: aerobic training; CG: control group; HVMIT: high-volume moderate-intensity training; HTG: high-volume training group; IG: intervention group; LVMIT: low-volume moderate-intensity training; MICT: moderate-intensity continuous training; MTG: moderate volume training group; P-HIT: high-intensity training with passive recovery; RT: resistance training; SD: standard deviation; TH: training high resting cardiac vagal modulation; TL: training low resting cardiac vagal modulation; 95 % CI: 95% confidence interval. The figure was constructed by the authors of this article using RevMan 5.4 software.

In the time-domain analysis, data from the 14-unit analysis were pooled to measure the changes in SDNN (227 in the intervention group (IG) and 147 in the control group (CG)), and RMSSD (198 participants in the intervention group and 150 in the control group). The analysis showed significant improvements in favor of the IG (SMD: 0.58 (0.16, 1.00); Test for overall effect: Z = 2.70 (p = 0.007); Figure 2 (a), and SMD: 0.84 (0.36, 1.31); Test for overall effect: Z = 3.46 (p = 0.0005); Figure 2 (b), in SDNN and RMSSD, respectively. However, changes in pNN50, using a 7-unit-analysis (113 in IG and 83 in CG) were not significant between the intervention and control groups. (p > 0.05; Figure 2c).

**Figure 3:**
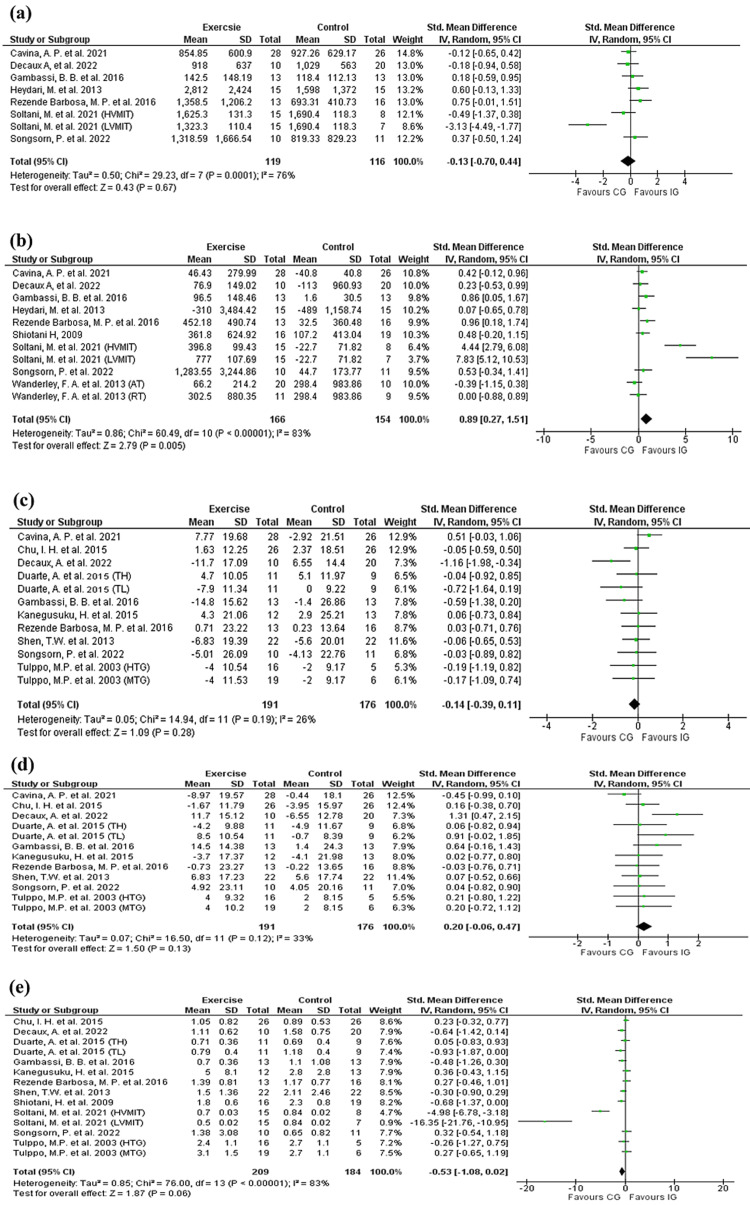
Forest plots of the pooled standardized mean difference (SMD) between exercise and control groups for frequency-domain HRV domains. (a) LF  [35,37,40,42,44,45,48]; (b) HF [35,37,40,42,44,45,47-49], (c) LFnu [15,35,36,38,41-45,48], (d) HFnu [15,35,36,38 41-45,48], (e) LF/HF [15,36,38,40-45,47,48] AT: aerobic training; CG: control group; HVMIT: high-volume moderate-intensity training; HTG: high-volume training group; IG: intervention group; LVMIT: low-volume moderate-intensity training; MTG: moderate-volume training group; RT: resistance training; SD: standard deviation; TH: training high resting cardiac vagal modulation; TL: training low resting cardiac vagal modulation; 95% CI: 95% confidence interval. The figure was constructed by the authors of this article using RevMan 5.4 software.

Analysis of frequency-domain HRV indices showed significant improvement in HF (using 11-unit analysis (166 in IG, 154 in CG)) in favor of the exercise group (SMD: 0.89 (0.27, 1.51); test for overall effect: Z = 2.79 (p = 0.005); (Figure 3b), and no significant effect was detected for the rest of the indices (HFnu, LF, LFnu, and LF/HF) (p > 0.05) (Figures 3a,3c-3e).

Substantial significant heterogeneity was observed in the SDNN, RMSSD, HF, LF, and LF/HF indices (I² > 50%; p < 0.0001), which further supports our choice to use a random-effects model. Low heterogeneity was identified in LFnu and HFnu (I² = 26% and 33%, respectively; p > 0.05) and no heterogeneity was reached in pNN50, (I² = 0 %; p > 0.05).

**Figure 4:**
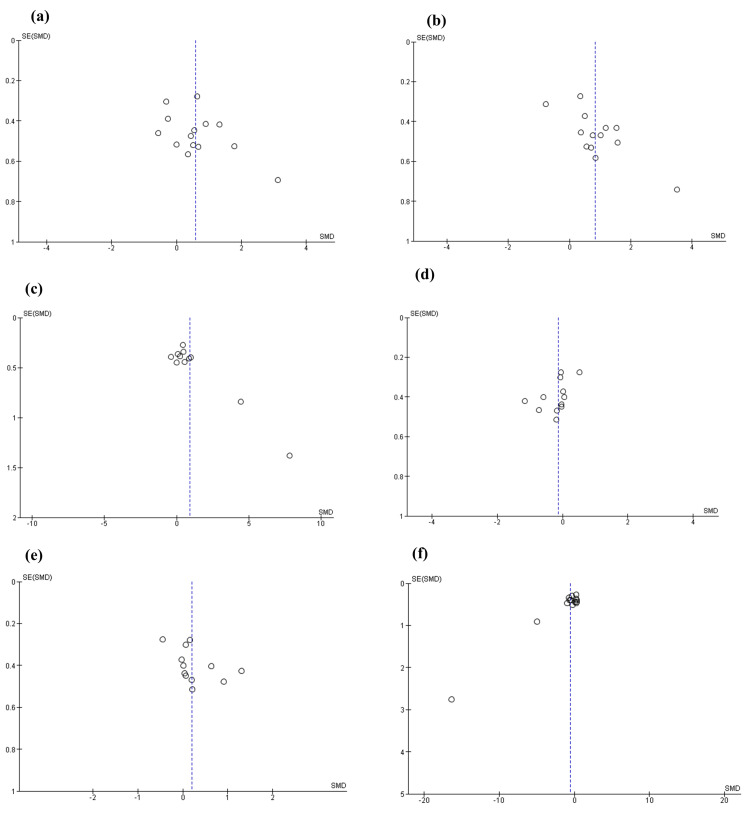
Funnel plots’ summary for publication bias detection for SDNN (a), RMSSD (b), HF (c), LFnu (d), HFnu (e), and LF/HF (f). X-Axis: standardized mean difference (SMD); Y-Axis: standard error (SE). SDNN: standard deviation of normal to normal; RMSSD: root mean square of successive RR-intervals differences; HF: power of the high-frequency band; LFnu: normalized unit of power of the low-frequency band; HFnu: normalized unit of power of the high-frequency band The figure was constructed by the authors of this article using RevMan 5.4 software.

The symmetrical shape of the separate funnel plots of the included studies suggests that there was no clear risk of publication bias or small-study effect. For the HF and LF/HF ratio parameters, funnel plots showed a scatter of two plots corresponding to two datasets indicating heterogeneity (when eliminated from the analysis, heterogeneity became non-substantial). Moreover, the plot distribution was very narrow at the top of the plot, showing no publication bias or a small-study effect. Given that funnel plots are inappropriate when the number of individual studies is less than 10), asymmetry was not evaluated for pNN50 and LF parameters (Figure 4).

According to the subgroup analyses performed, a statistically significant subgroup effect (p = 0.04) was identified in the influence of age on the exercise-induced effect on HF, indicating that age significantly modifies the effect of intervention in comparison to the CG. The effect favored the IG over the CG for both age groups, although the intervention effect was greater for the group of participants aged ≥ 40 years than for the group of participants aged < 40 years; therefore, the subgroup effect was quantitative [50]; SMD: 0.89 (0.27, 1.51); p = 0.03, VS. SMD: 0.30 (0.04, 0.57); p = 0.02. However, it is important to acknowledge the uncertainty of the evidence because of the high heterogeneity identified within the subgroup. Similarly, the results of the subgroup analysis based on age for the LF/HF ratio showed a significant quantitative reduction, which was higher in the group of participants aged ≥ 40 years than in those aged 40 years (SMD: -2.39 (-4.21, -0.56); p = 0.01 VS. SMD: -0.13 (-0.45, 0.19), respectively; (p > 0.05). However, it is important to acknowledge the uncertainty in the evidence owing to the substantial heterogeneity identified within this subgroup. The subgroup analysis performed to test whether sex modified the effect of the intervention showed a significant result in HF and a non-significant effect in the remaining parameters. In HF, males showed higher improvement compared to females and to the studies conducted on a group of both sexes. Of note, the effect was non-significant among the latter (SMD: 2.03 (0.54, 3.51); p = 0.008, VS. SMD: 0.91 (0.35, 1.47); p = 0.001, VS. SMD: 0.16 (-0.27, 0.60); p = 0.46). Again, heterogeneity was substantial in the male group, indicating that the analysis was unlikely to produce certain conclusions. Finally, the test for the type of exercise (aerobic, anaerobic, and combined) suggested that there is a statistically significant subgroup effect (p = 0.04) on LF, meaning that the type of exercise significantly modifies the effect of exercise in comparison to the control. Exercise was favored for the anaerobic and combined exercise groups, and inversely for the aerobic exercise group. Hence, the subgroup effect was qualitative [50]. Subgroup analyses based on the remaining potential influencing factors did not reveal any statistically significant effects (p > 0.05) (Table 2).

**Table 2:** Subgroup analyses for HRV parameters. Chi²: Chi-squared statistic for subgroup difference; HF: power of the high-frequency band; HFnu: normalized unit of power of the high-frequency power; I²: heterogeneity index (%); LF: power of the low-frequency band; LFnu: normalized unit of power of the low-frequency band; LF/HF: ratio of low frequency to high frequency; K (IG, CG): number of datasets in each subgroup (number of participants in the intervention group, number of participants in the control group); N/A: not applicable; pNN50: percentage of adjacent NN intervals varying by more than 50 milliseconds; RMSSD: root mean square of successive RR-intervals differences; SMD: standardized mean value; SDNN: standard deviation of NN intervals; 95% CI: 95% confidence interval A p-value of < 0.05 was considered statistically significant.

Variable	Subgroup	K (IG, CG)	SMD (95% CI)	Chi²	p-value	I² (%)
SDNN						
Age	< 40 years	7 (117, 83)	0.39 (-0.07, 0.84)	0.95	0.33	54
	≥ 40 years	7 (120, 72)	0.85 (0.04, 1.65)			81
Sex	Males	8 (138, 66)	0.86 (0.30, 1.42)	2.64	0.27	63
	Females	4 (59, 60)	0.33 (-0.56, 1.21)			81
	Both sexes	2 (30, 21)	0.11 (-0.67, 0.89)			44
Type of exercise	Aerobic	8 (160, 97)	0.71 (0.07, 1.34)	4.73	0.09	79
	Anaerobic	5 (64, 42)	0.26 (-0.26, 0.78)			36
	Combined	1 (13, 16)	1.33 (0.51, 2.15)			N/A
Duration of training program	< 12 weeks	4 (67, 44)	0.18 (-0.29, 0.66)	2.15	0.14	25
	≥ 12 weeks	10 (160, 103)	0.73 (0.18, 1.28)			74
RMSSD						
Age	< 40 years	6 (88, 86)	0.69 (0.33, 1.06)	0.37	0.54	24
	≥ 40 years	7 (110, 64)	1.01 (0.06, 1.95)			85
Sex	Males	9 (140, 88)	0.87 (0.39, 1.35)	0.18	0.91	60
	Females	3 (48, 51)	0.63 (-0.90, 2.16)			92
	Both sexes	1 (10, 11)	1.01 (0.09, 1.93)			N/A
Type of exercise	Aerobic	7 (127, 94)	0.78 (-0.04, 1.60)	0.17	0.92	86
	Anaerobic	4 (53, 33)	0.97 (0.49, 1.46)			0
	Combined	2 (28, 31)	0.99 (-0.04, 2.02)			71
Duration of training program	< 12 weeks	2 (32, 33)	0.08 (-1.66, 1.83)	0.92	0.34	90
	≥ 12 weeks	11 (166, 117)	0.96 (0.55, 1.38)			58
pNN50						
Age	< 40 years	2 (33, 34)	0.23 (-0.25, 0.71)	0.03	0.86	0
	≥ 40 years	5 (80, 49)	0.17 (-0.31, 0.64)			0
Sex	Males	4 (60, 29)	0.32 (-0.15, 0.78)	0.53	0.77	0
	Females	2 (35, 35)	0.07 (-0.70, 0.84)			N/A
	Both sexes	1 (18, 19)	0.06 (-0.59, 0.70)			N/A
Type of exercise	Aerobic	3 (55, 46)	0.19 (-0.35, 0.74)	0.69	0.71	0
	Anaerobic	3 (43, 22)	0.07 (-0.47, 0.60)			0
	Combined	1 (15, 15)	0.45 (-0.28, 1.17)			N/A
Duration of training program	< 12 weeks	2 (40, 41)	0.06 (-0.59, 0.70)	0.25	0.62	N/A
	≥ 12 weeks	5 (73, 42)	0.25 (-0.15, 0.65)			0
LF						
Age	< 40 years	5 (76, 88)	0.24 (-0.14, 0.62)	2.28	0.13	29
	≥ 40 years	3 (43, 28)	-1.06 (-2.70, 0.59)			88
Sex	Males	4 (73, 56)	-0.64 (-1.76, 0.47)	3.27	0.19	87
	Females	2 (26, 29)	0.47 (-0.09, 1.03)			7
	Both sexes	2 (20, 31)	0.06 (-0.51, 0.63)			0
Type of exercise	Aerobic	3 (58, 41)	-1.12 (-2.56, 0.32)	6.31	0.04	88
	Anaerobic	3 (33, 44)	0.10 (-0.36, 0.56)			0
	Combined	2 (28, 31)	0.67 (0.14, 1.20)			0
Duration of training program	< 12 weeks	2 (20, 31)	0.06 (-0.51, 0.63)	0.35	0.55	0
	≥ 12 weeks	6 (99, 85)	-0.23 (-0.99, 0.53)			82
HF						
Age	< 40 years	8 (123, 126)	0.30 (0.04, 0.57)	4.06	0.04	6
	≥ 40 years	3 (43, 28)	4.20 (0.42, 7.97)			94
Sex	Males	5 (83, 76)	2.03 (0.54, 3.51)	8.41	0.01	92
	Females	2 (26, 29)	0.91 (0.35, 1.47)			0
	Both sexes	4 (57, 49)	0.16 (-0.27, 0.60)			17
Type of exercise	Aerobic	5 (94, 70)	1.99 (0.48, 3.50)	3.92	0.14	93
	Anaerobic	4 (44, 53)	0.41 (-0.00, 0.83)			0
	Combined	2 (28, 31)	0.50 (-0.38, 1.38)			64
Duration of training program	< 12 weeks	3 (36, 50)	0.41 (-0.03, 0.85)	2.53	0.11	0
	≥ 12 weeks	8 (130, 104)	1.23 (0.32, 2.13)			88
LFnu						
Age	< 40 years	9 (144, 128)	-0.14 (-0.48, 0.19)	1.54	0.21	40
	≥ 40 years	3 (47, 48)	-0.94 (-2.16, 0.27)			86
Sex	Males	6 (97, 68)	-0.45 (-1.22, 0.32)	1.11	0.57	79
	Females	4 (74, 77)	-0.13 (-0.45, 0.19)			0
	Both sexes	2 (20, 31)	-0.60 (-1.71, 0.50)			71
Type of exercise	Aerobic	7 (133, 103)	0.00 (-0.26, 0.27)	4.57	0.10	3
	Anaerobic	4 (45, 57)	-1.00 (-1.89, -0.11)			76
	Combined	1 (13, 16)	0.03 (-0.71, 0.76)			N/A
Duration of training program	< 12 weeks	6 (103, 90)	-0.23 (-0.56, 0.10)	0.33	0.57	15
	≥ 12 weeks	6 (88, 86)	-0.47 (-1.20, 0.26)			80
HFnu						
Age	< 40 years	9 (144, 128)	0.22 (-0.14, 0.57)	0.00	0.97	46
	≥ 40 years	3 (47, 48)	0.20 (-0.20, 0.61)			0
Sex	Males	6 (97, 68)	0.06 (-0.32, 0.44)	0.93	0.63	25
	Females	4 (74, 77)	0.18 (-0.15, 0.50)			0
	Both sexes	2 (20, 31)	0.68 (-0.57, 1.92)			77
Type of exercise	Aerobic	7 (133, 103)	0.07 (-0.22, 0.36)	1.84	0.40	12
	Anaerobic	4 (45, 57)	0.50 (-0.09, 1.09)			52
	Combined	1 (13, 16)	-0.03 (-0.76, 0.71)			N/A
Duration of training program	< 12 weeks	6 (103, 90)	0.30 (-0.06, 0.65)	0.39	0.53	27
	≥ 12 weeks	6 (88, 86)	0.12 (-0.29, 0.53)			42
LF/HF						
Age	< 40 years	9 (132, 121)	-0.13 (-0.45, 0.19)	5.66	0.02	32
	≥ 40 years	5 (77, 63)	-2.39 (-4.21, -0.56)			94
Sex	Males	7 (99, 57)	-1.56 (-2.97, -0.15)	4.64	0.10	91
	Females	4 (74, 77)	-0.04 (-0.40, 0.31)			16
	Both sexes	3 (36, 50)	-0.38 (-0.98, 0.23)			46
Type of exercise	Aerobic	9 (151, 111)	-1.02 (-1.90, -0.13)	4.93	0.08	88
	Anaerobic	4 (45, 57)	-0.13 (-0.64, 0.39)			39
	Combined	1 (13, 16)	0.27 (-0.46, 1.01)			N/A
Duration of training program	< 12 weeks	8 (131, 116)	-0.26 (-0.57, 0.05)	3.08	0.08	24
	≥ 12 weeks	6 (78, 68)	-1.68 (-3.24, -0.12)			93

Risk of bias assessment

**Figure 5:**
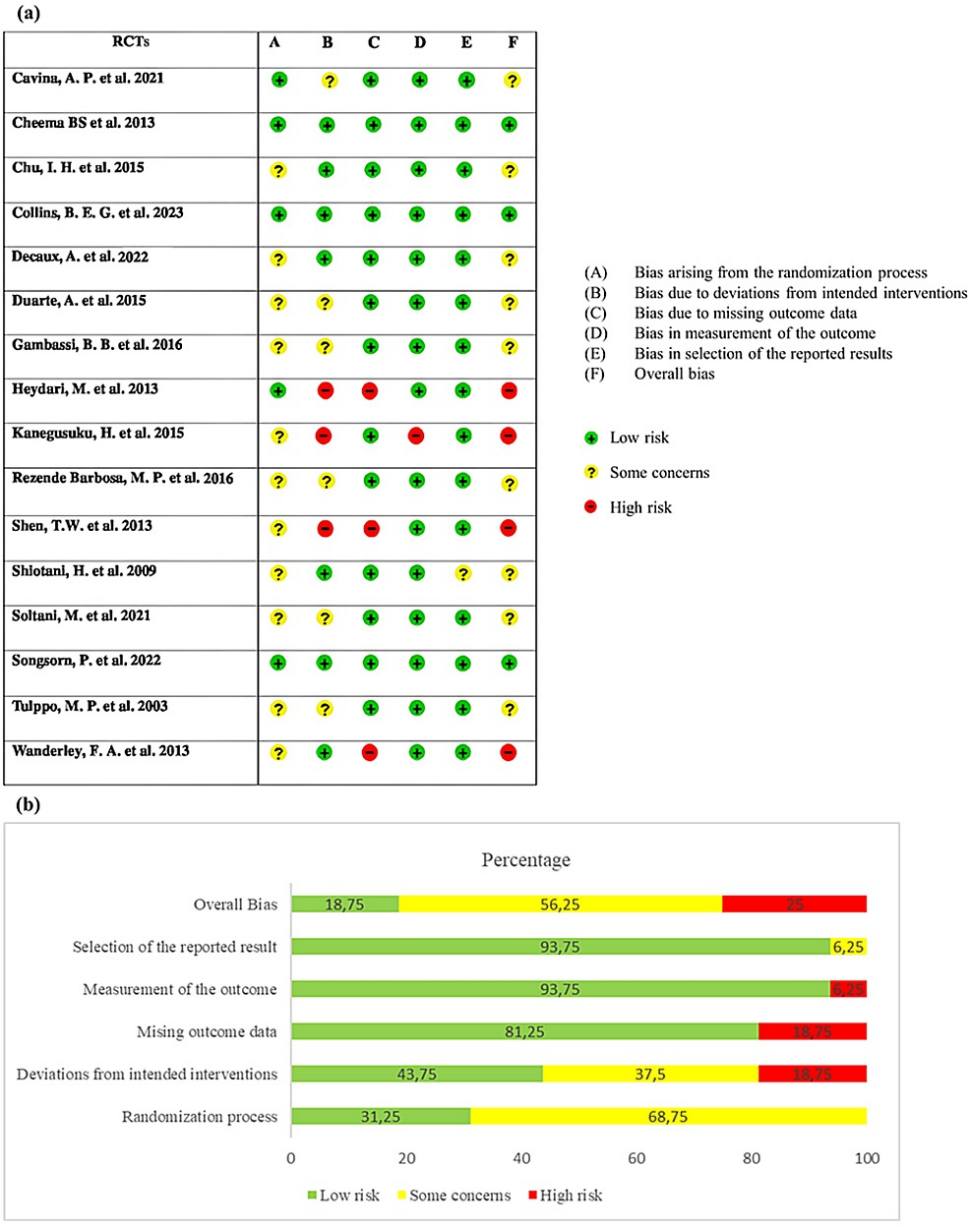
Results of risk of bias assessment using the Cochrane risk of bias 2 (RoB2) tool. Risk of bias assessment (a) and the percentage of studies presenting each risk of bias level in each domain (b) [15,35-49]. The figure was constructed by the authors of this article using RevMan 5.4 software.

The risk of bias in the included studies was evaluated using Rob2 and TESTEX tools. The results are shown in Figures 5a-5b and Table 3, respectively. According to the Rob2 evaluation results, three of the 16 studies reviewed had a low risk of bias [39,46,48], nine were judged as raising some concerns [15,35,36,40,42-45,47], and four were rated as having a high risk of bias [35,38,41,49].

Analysis of each Rob2 domain revealed that 68.75% of the included studies were rated as having some concerns with the randomization process, and 31.25% were at a low risk of bias due to inadequate randomization. Regarding risk related to deviation from the intended intervention (assignment-to-intervention), 43.75% of the studies had low risk, while 37.5% and 18.75% had some concerns and high risk, respectively. In terms of missing outcome data, 81.25% of the trials had a low risk of bias, whereas 18.75% had a high risk of bias. Of the studies, 93.75% were at low risk related to the measurement of the outcome domain, and only 6.25 were at high risk. Regarding the selection of the reported result-related risk, 93.75% of the studies reported a low risk of bias, whereas 6.25% showed some concerns. The overall risk of bias assessment showed that 56.25% of the included studies had some concerns, 25% had a high risk of bias, and 18.75% had a low risk of bias (Figure 5b).

Using the TESTEX scale, the overall mean score of the reviewed studies was 9.2 ± 2.1 (min: 6, max: 13). No study was judged to have poor methodological quality (score less than 6), two were rated as excellent (scored from 12 to 15 points) [39,46], nine as good (9 to 11 points) [15,35,37,42,43,45,47-49], and five studies were judged to have fair quality (6 to 8 points) [36,38,40,41,44]. Out of the 16 RCTs included, 10 studies specified the randomization method used [15,35,37,39,40,43,46,47-49], five studies reported the use of allocation concealment [35,37,39,46,48], and only three studies blinded the outcome assessor [35,37,46]. Eighty-five percent or more of participants completed the intervention and were assessed before and after the end of the training, with attendance frequency and adverse events reported (3 points) in only two studies [45,46], while none of these three criteria were met in six studies [35,36,37,38,40,41]. Nine studies performed intention-to-treat analyses [15,35,39,42,43,45-48]. Only one study monitored physical activity in the CG during the intervention period [49]. The relative exercise intensity remained constant in six studies [35,39,40,42,44,49]. All reviewed trials reported eligibility criteria and clearly stated that exercise volume with participants was similar at baseline, except for Rezende Barbosa et al. [44], where the age of participants was significantly different between the intervention and control groups (Table 3).

**Table 3:** Methodological quality assessment of included studies judged using the TESTEX scale. Item 1: eligibility criteria specified; Item 2: randomization specified; Item 3: allocation concealment; Item 4: groups similar at baseline; Item 5: blinding of assessor (for at least one key outcome); Item 6: outcome measures assessed in 85% of participants; Item 7: intention-to-treat analysis; Item 8: between-group statistical comparisons reported; Item 9: point measures and measures of variability for all reported outcome measures; Item 10: activity monitoring in control groups; Item 11: relative exercise intensity remained constant; Item 12: exercise volume and energy expenditure stated; Overall: the overall TESTEX score for each included study; RCT: randomized controlled trial.

RCTs	Study quality					Study reporting							
	Item 1	Item 2	Item 3	Item 4	Item 5	Item 6	Item 7	Item 8	Item 9	Item 10	Item 11	Item 12	Overall
Cavina et al. 2021 [35]	1	1	1	1	1	0	1	2	1	0	1	1	11
Cheema et al. 2013 [46]	1	1	1	1	1	3	1	2	1	0	0	1	13
Chu et al. 2015 [43]	1	1	0	1	0	2	1	2	1	0	0	1	10
Collins et al. 2023 [39]	1	1	0	1	0	2	1	2	1	0	1	1	12
Decaux et al. 2022 [45]	1	0	0	1	0	3	1	2	1	0	0	1	10
Duarte et al. 2015 [15]	1	1	0	1	0	2	1	2	1	0	0	1	10
Gambassi et al. 2016 [42]	1	0	0	1	0	2	1	2	1	0	1	1	10
Heydari et al. 2013 [37]	1	1	1	1	1	0	0	2	1	0	0	1	9
Kanegusuku et al. 2015 [38]	1	0	0	1	0	0	0	2	1	0	0	1	6
Rezende Barbosa et al. 2016 [44]	1	0	0	0	0	2	0	2	1	0	1	1	8
Shen and Wen 2013 [41]	1	0	0	1	0	0	0	2	1	0	0	1	6
Shiotani et al. 2009 [47]	1	1	0	1	0	2	1	2	1	0	0	1	10
Soltani et al. 2021 [40]	1	1	0	1	0	0	0	2	1	0	1	1	8
Songsorn et al. 2022 [48]	1	1	0	1	0	2	1	2	1	0	0	1	11
Tulppo et al. 2003 [36]	1	0	0	1	0	0	0	2	1	0	0	1	6
Wanderley et al. 2013 [49]	1	1	0	1	0	2	0	2	1	1	1	1	11

Sensitivity analysis

To determine whether studies with a high risk of bias had any effect on the pooled outcome estimate, sensitivity analyses were conducted by removing trials with a high risk of bias, as judged by Rob2, from the primary overall analysis. The results obtained after excluding the Heydari et al. [37], Kanegusuku et al. [38], Shen and Wen [41] and Wanderley et al. [49] studies from the overall analysis of the effect of training on HRV showed differences in the ES which improved after excluding high risk of bias studies for SDNN (SMD from 0.58 (0.16, 1.00) to 0.86 (0.46, 1.27)), RMSSD (SMD from 0.84 (0.36, 1.31) to 1.02 (0.60, 1.45)), and HF (SMD from 0.89 (0.27, 1.51) to 1.37 (0.55, 2.19), and decreased and became significant for LF/HF (SMD from -0.53 (-1.08, 0.02) to -0.69 (-1.36, -0.02).

However, the magnitude of heterogeneity remained substantial in SDNN, RMSSD, and HF analyses, and it only diminished when we excluded studies with a high risk of bias and outliers (those not evenly distributed around the funnel plot). For SDNN, heterogeneity diminished from I² = 70% (p < 0.0001) to I² = 0% (P = 0.74), for RMSSD, it diminished from I² = 74% (p < 0.00001) to I² = 0% (p = 0.48), and for HF from I² = 83% (p < 0.00001) to I² = 28% (p = 0.19). The details of the sensitivity analysis are presented in Table 4.

**Table 4:** Summary of sensitivity analyses. Each HRV parameter summary of meta-analysis is presented in three conditions: 1) the primary overall model with all the included studies; 2) after exclusion of low methodological quality studies; 3) in addition to condition 2), outliers (studies not evenly distributed around the funnel plot) are excluded. A-HIT: high-intensity training with active recovery; AT: aerobic training; CG: pooled number of participants in the control group; HF: power of the high-frequency band; HFnu: normalized unit of power of the high-frequency band; HTG: high-volume training group; HVMIT: high-volume moderate-intensity training; IG: pooled number of participants in the intervention group; LF: power of the low-frequency band; LFnu: normalized unit of power of the low-frequency band; LVMIT: low-volume moderate-intensity training; MICT: moderate-intensity continuous training; MTG: moderate volume training group; P-HIT: high-intensity training with passive recovery; pNN50: percentage of adjacent NN intervals varying by more than 50 milliseconds; RT: resistance training; RMSSD: root mean square of successive RR-intervals differences; SDNN: standard deviation of NN intervals; TH: training high resting cardiac vagal modulation; TL: training low resting cardiac vagal modulation; 95 % CI: 95% confidence interval. A p-value of < 0.05 was considered statistically significant.

HRV parameter	Primary meta-analysis results		Meta-analysis results after removal of high risk of bias trials		Meta-analysis results after removal of high risk of bias trials and outliers		
							Overall effect size SMD (95% CI)(Test result for overall effect size)	Heterogeneity I² index (Chi² test result)	Overall effect size SMD (95% CI) (Test result for overall effect size)	Heterogeneity I² index (Chi² test result)	Overall effect size SMD (95% CI) (Test result for overall effect size)	Heterogeneity I² index (Chi² test result)		
							SDNN	0.58 (0.16, 1.00)	I² = 70%	0.86 (0.46, 1.27)	I² = 52%	0.64 (0.36, 0.93)	I² = 0%		
	(p = 0.007)	(p < 0.0001)	(p < 0.0001)	(p = 0.02)	(p < 0.00001)	(p = 0.74)			
IG: 227		IG: 174		IG: 144				
CG: 147		CG: 106		CG: 91				
		Removed studies		Removed studies				
		Shen and Wen 2013 [41]		Shen and Wen 2013 [41]				
		Wanderlay et al. 2013 [49]		Soltani et al. 2021 [40]				
				Wanderlay et al. 2013 [49]				
RMSSD	0.84 (0.36, 1.31)	I² = 74%	1.02 (0.60, 1.44)	I² = 56%	0.75 (0.47, 1.04)	I² = 0%		
	(p = 0.0005)	(p < 0.00001)	(p < 0.00001)	(p = 0.01)	(p < 0.00001)	(p = 0.48)		
	IG: 198		IG: 161		IG: 131			
	CG: 150		CG: 113		CG: 98			
			Removed studies		Removed studies			
			Shen and Wen 2013 [41]		Shen and Wen 2013 [41]			
					Soltani et al. 2021 [40]			
pNN50	0.20 (-0.14, 0.54)	I² = 0%	0.13 (-0.25, 0.51)	I² = 0%	--	--		
	(p = 0.25)	(p = 0.89)	(p = 0.51)	(p = 0.90)				
	IG: 113		IG: 76					
	CG: 83		CG: 47					
			Removed studies					
			Heydari et al. 2013 [37]					
			Shen and Wen 2013 [41]					
LF	-0.13 (-0.70, 0.44)	I²= 76%	-0.24 (-0.88, 0.39)	I² = 77%	0.14 (-0.19, 0.48)	I² = 8%		
	(p = 0.67)	(p = 0.0001)	(p = 0.45)	P = (0.0002)	(p = 0.40)	(p = 0.36)		
	IG: 119		IG: 104		IG: 74			
	CG: 116		CG: 101		CG: 86			
			Removed studies		Removed studies			
			Heydari et al. 2013 [37]		Heydari et al. 2013 [37]			
					Soltani et al. 2021 [40]			
HF	0.89 (0.27, 1.51)	I² = 83%	1.37 (0.55, 2.19)	I² = 86%	0.55 (0.26, 0.84)	I² = 0%		
	(p = 0.005)	(p < 0.00001)	(p = 0.001)	(P < 0.00001)	(p = 0.0002)	(p = 0.77)		
	IG: 166		IG: 120		IG: 90			
	CG: 154		CG: 120		CG: 105			
			Removed studies		Removed studies			
			Heydari et al. 2013 [37]		Heydari et al. 2013 [37]			
			Shen and Wen 2013 [41]		Shen and Wen 2013 [41]			
			Wanderlay et al. 2013 [49]		Soltani et al. 2021 [40]			
					Wanderlay et al. 2013 [49]			
LFnu	-0.14 (-0.39, 0.11)	I² = 26%	-0.19 (-0.50, 0.12)	I² = 39%	-0.04 (-0.30, 0.21)	I² = 2%		
	(p = 0.28)	(p = 0.19)	(p = 0.24)	(p = 0.10)	(p = 0.73)	(p = 0.42)		
	IG: 191		IG: 157		IG: 147			
	CG: 176		CG: 141		CG: 121			
			Removed studies		Removed studies			
			Shen and Wen 2013 [41]		Decaux et al. 2022 [45]			
					Shen and Wen 2013 [41]			
HFnu	0.20 (-0.06, 0.47)	I² = 33%	0.26 (-0.07, 0.58)	I² = 44%	0.11 (-0.15, 0.37)	I² = 10%		
	(p = 0.12)	(p = 0.12)	(p = 0.13)	(p = 0.06)	(p = 0.41)	(p = 0.36)		
	IG: 191		IG: 157		IG: 147			
	CG: 176		CG: 141		CG: 121			
			Removed studies		Removed studies			
			Kanegusuku et al. 2015 [38]		Decaux et al. 2022 [45]			
			Shen and Wen 2013 [41]		Kanegusuku et al. 2015 [38]			
					Shen and Wen 2013 [41]			
LF	-0.13 (-0.70, 0.44)	I²= 76%	-0.24 (-0.88, 0.39)	I² = 77%	0.14 (-0.19, 0.48)	I² = 8%		
	(p = 0.67)	(p = 0.0001)	(p = 0.45)	P = (0.0002)	(p = 0.40)	(p = 0.36)		
	IG: 119		IG: 104		IG: 74			
	CG: 116		CG: 101		CG: 86			
			Removed studies		Removed studies			
			Heydari et al. 2013 [37]		Heydari et al. 2013 [37]			
					Soltani et al. 2021 [40]			
HF	0.89 (0.27, 1.51)	I² = 83%	1.37 (0.55, 2.19)	I² = 86%	0.55 (0.26, 0.84)	I² = 0%		
	(p = 0.005)	(p < 0.00001)	(p = 0.001)	(P < 0.00001)	(p = 0.0002)	(p = 0.77)		
	IG: 166		IG: 120		IG: 90			
	CG: 154		CG: 120		CG: 105			
			Removed studies		Removed studies			
			Heydari et al. 2013 [37]		Heydari et al. 2013 [37]			
			Wanderley et al. 2013 [49]		Soltani et al. 2021 [40]			
					Wanderley et al. 2013 [49]			
LF/HF	-0.53 (-1.08, 0.02)	I² = 83%	-0.69 (-1.36, -0.02)	I² = 85%	-0.17 (-0.46, 0.13)	I² = 28%		
	(p = 0.06)	(p < 0.00001)	(p = 0.04)	(p < 0.00001)	(p = 0.26)	(p = 0.19)		
	IG: 209		IG: 175		IG: 145			
	CG: 184		CG: 149		CG: 134			
			Removed studies		Removed studies			
			Kanegusuku et al. 2015 [38]		Kanegusuku et al. 2015 [38]			
			Shen and Wen 2013 [41]		Shen and Wen 2013 [41]			
					Soltani et al. 2021 [40]			

Discussion

In response to the research question of this meta-analysis, the findings showed that exercise training improves the SDNN and RMSSD time-domain parameters reflecting overall ANS activity and parasympathetic modulation, respectively, in healthy adults. With regard to the frequency-domain HRV, the effect of exercise training was significant on the HF parameter, which is a marker of parasympathetic system activity.

Recent systematic reviews conducted among healthy adults highlighted the benefits of different types of exercise training, including aerobic, resistance, coordinative, high-intensity, and multimodal interventions on HRV, and found that higher training intensities and frequencies are more likely to improve HRV parameters [51,52]. However, these results were qualitative in nature and did not provide a meta-analysis of the studies’ findings to quantitatively identify the intervention-induced effects on various HRV parameters. Our findings showed an important improvement in RMSSD in favor of the IG compared to the CG, with an SMD of 0.84 (0.36, 1.31); (p = 0.0005). In line with this result, a meta-analysis addressing the impact of exercise training on cardiac-parasympathetic activity in sedentary individuals reported an SMD increase of 0.57 (95% CI = 0.23, 0.91) in the RMSSD index [53]. Notably, the previous meta-analysis [53] was conducted on RCTs and non-RCTs. Although the subgroup analysis based on the study design (randomization/no randomization) was not statistically significant, an uneven covariate distribution of studies between the groups was observed, which may have prevented the analysis from showing a potential effect. Conversely, our meta-analysis considered only RCTs.

Improvements in HRV parameters have also been demonstrated in diseased populations following regular physical activity programs. Meta-analyses investigating the effect of exercise training on patients with type 2 diabetes [54], coronary artery disease [55], and heart failure [56] showed a significant increase in RMSSD in intervention groups. The SMD was 0.62 (0.28, 0.95) for the type 2 diabetes meta-analysis, 0.30 (0.12, 0.49) for coronary artery disease, and in the meta-analysis of heart failure [54] the mean difference (MD) was used instead of the SMD and was about 10.44 (0.60, 20.28). Comparing the ES of exercise training induced between our healthy population and the abovementioned diseased populations, the SMD reported in our meta-analysis was the highest. However, given the differences in health conditions and the number and type of trials used in each meta-analysis, such comparisons should be taken with caution.

Moreover, the present meta-analysis showed a significant improvement in SDNN (SMD: 0.58 (0.16, 1.00)) in favor of the exercise training group. Similarly, Picard et al. [54] found in their meta-analysis an improvement of 0.59 (0.26, 0.93) in SDNN value. Nonetheless, their analysis involved patients with type 2 diabetes, whereas our meta-analysis included healthy subjects. Our analysis of the pNN50 did not reveal any significant results. It should be noted that this analysis included 7-unit analyses, which may not be sufficient to detect the pooled exercise-induced effect. Moreover, our analysis of pNN50 was performed using final values (post-intervention) rather than changes from baseline, and this was for the sake of precision when dealing with missing standard deviations of the difference in mean, as recommended by the Cochrane Handbook [32].

Regarding frequency-domain HRV, the results showed a significant improvement in the absolute value of HF, and no significant effect was detected in the normalized value of HF. Similarly, findings from the meta-analysis of Casanova-Lizón et al. [53] on the effect of exercise training on HF in sedentary healthy adults were statistically significant and yielded an SMD of 0.23 (0.00, 0.46); p = 0.05, VS. and SMD of 0.89 (0.27, 1.51), p = 0.005, found in our meta-analysis. Notably, in the meta-analysis by Casanova-Lizón et al. [53], the analysis included the absolute values of HF and excluded normalized units of HF, which may explain the comparable results between our meta-analysis, where absolute and normalized HF values were investigated separately, and the aforementioned meta-analysis [53]. The findings of our pooled analyses did not show any significant effect (p > 0.05) on LF (in both absolute and normalized units) and LF/HF ratio indices.

Of note, the normalized spectral values present a set of redundancies with respect to each other, as well as to the LF/HF ratio. LFnu and HFnu are highly linearly related as LFnu = 1-HFnu [57,58]. This implies that changes in one imply changes in the other (their sum is 1) [57,58], which is not true for absolute values, as it is possible to simultaneously increase and decrease. Moreover, considering the collinearity between the normalized indices (LF and HF), the statistical significance of one may equivalently imply the statistical significance of the other [57,58]. Taking into account these mathematical interpretations of normalized spectral HRV indices, this may explain why frequency-domain HRV in normalized units, in addition to the LF/HF ratio, were all non-significant in the present meta-analysis. These conclusions support the importance of reporting results of absolute values of frequency-domain HRV, and our decision to analyze absolute and normalized values separately.

The impact of exercise training on frequency-domain HRV was also examined through a meta-analysis conducted in patients with coronary artery disease [55], which, in contrast to our results, reported a non-significant impact on HF, but a significant effect on LF and LF/HF. However, in the HF analysis, the authors included values measured in absolute, logarithmically transformed, and normalized units. Notably, HF and RMSSD are both vagal-related HRV indices [9], and our findings yielded a significant effect of the intervention on both parameters, which may confirm the exercise-induced effect on parasympathetic activity in our target population.

HRV measures have been extensively influenced by various factors. Hence, we conducted subgroup analyses to estimate the impact of the interventions on the subgroups of participants based on their characteristics and those of the intervention used. Such analyses may also be used to investigate the sources of heterogeneity. Subgroup analyses based on participant age identified significant differences in the HF and LF/HF indices. Age-related changes in HRV have been reported in several studies [59-63]. Importantly, it has been shown that ANS responses to exercise may not be limited by aging [59], and enhanced vagal modulation following aerobic exercise training has been reported in adults aged up to 68 years old [27,64,65]. Moreover, in middle-aged individuals, it has been observed that regular physical activity is associated with improved HRV measures, particularly those related to increased vagal modulation and decreased sympathetic activity [66,67]. Other subgroup analyses showed a significant effect of sex and type of exercise training on the HF and LF power of HRV, respectively. Notably, in most of the subgroup analyses conducted, there was an uneven distribution of studies between subgroups, which may cast doubt on the power of these analyses and mean that subgroup differences could not be detected [50]. A meta-analysis conducted by Casanova-Lizón et al. [53] showed that the effects of sex, type and length of intervention, and number of sessions on RMSSD and HF were not significant. Conversely, in patients with type 2 diabetes, a meta-analysis revealed that the type of exercise may influence the effect of training on the LF/HF ratio, with improvements after endurance or resistance training compared with combined training. In addition, increased pNN50 was observed after endurance training compared with resistance and combined training [54]. Similarly, a systematic review of trials involving healthy older adults reported beneficial effects of different exercise interventions on cardiac autonomic modulation, with the exception of anaerobic training. Consistently, in another meta-analysis conducted by Bhati et al. [68], resistance training did not improve the vagal-related HRV indices in healthy individuals.

Sensitivity analyses were performed to address the substantial heterogeneity observed by removing the studies with a high risk of bias. Four studies were judged to have a low methodological quality. Thus, they were excluded from the analysis, leading to slight improvements in the parameters for which significant results were found in the primary meta-analysis. However, the heterogeneity was not fully explained; although it showed a slight decrease, it was still substantial. These findings support the decision to include these studies, despite their low methodological quality. In addition to the previous analyses, we excluded studies that were not evenly distributed around the funnel plot; thus, low or no heterogeneity was observed. In general, heterogeneity in studies of HRV has been widely discussed in the literature and is mostly related to the lack of standardized methodologies. However, in the present systematic review and meta-analysis, data were combined from the same study design (RCTs) with no disparate sample sizes. Moreover, these studies used validated HRV measurement devices, and the majority used the supine position when recording the HRV. Potential disparities may be attributed to breathing patterns due to the sensitivity of some HRV metrics to respiration frequency, especially in the HF band [69]. Of note, only two of the included studies used controlled breathing and were set at 12 cycles per minute. In the remaining studies, participants breathed spontaneously in seven trials, and the remaining seven trials did not report any information regarding respiration. The recording duration of a series of R-R intervals is also known to be heterogeneous among studies [70]. The duration used in our included studies varied from 5 min to 24h, with the most used durations being 5 min and 20 min, and 24h was used in only two trials. Notably, this limited number of studies has prevented the possibility of splitting pooled data into subgroups according to respiratory patterns and duration of recording.

To the best of our knowledge, this is the first systematic review and meta-analysis of RCTs investigating the effects of exercise training on time- and frequency-domain HRV parameters, in healthy adults. Our analysis considered participant (sex and age) and intervention (type and duration of the intervention) variables to determine potential effect-modifying factors and address sources of heterogeneity. Furthermore, small-study effect or publication bias, which is a threat to systematic reviews and meta-analyses, has not been identified. However, some limitations of this study should be considered when interpreting the findings. The limited number of RCTs included and the uneven distribution of studies between subgroups may have prevented the power of subgroup analyses. Moreover, substantial heterogeneity was observed in some of the HRV indices analyzed, which could not be fully explained by subgroup analyses. However, sensitivity analyses showed a significant decrease.

## Conclusions

The present meta-analysis provides convincing evidence of the effects of exercise training on HRV, focusing on both time- and frequency-domain parameters. The results indicated a significant and positive exercise training-induced effect on HRV parameters, mainly SDNN, reflecting overall autonomic activity, and RMSSD and HF, reflecting vagally mediated activity. These improvements suggest enhanced adaptation of ANS activity in response to exercise training. These results may not only underscore the benefits of exercise on HRV but also hint at a potential link between the exercise-induced effect on ANS modulation and the acknowledged positive effects on both physical and mental health. Nonetheless, it is worth noting that further investigations regarding the variation in this effect over time and among various populations are warranted.
